# Dual formation mechanisms of acidic variants in cysteine-engineered antibodies and strategies for their reduction

**DOI:** 10.3389/fbioe.2025.1615263

**Published:** 2025-10-23

**Authors:** Shasha Zhou, Xinyu Cao, Xuefei Yin, Jiawen Xu, Kai Gao, Zhenshou Wang

**Affiliations:** Shanghai Asymchem Biotechnology Co., Ltd, Shanghai, China

**Keywords:** THIOMABs, CHO cell culture, acidic variants, GSH capping, traditional PTM, dual formation mechanism

## Abstract

Cysteine-engineered antibodies (THIOMABs) are pivotal in the site-specific conjugation for ADC therapeutics. In this study, THIOMABs were expressed in CHO cell culture and showed a significantly higher proportion of acidic variants compared to traditional antibodies. A dual formation mechanism for acidic species was identified: glutathione (GSH) capping at engineered cysteine sites and traditional post-translational modifications (PTMs) during cell culture. Moreover, it was found that these two mechanisms exhibited overlapping effects on the formation of acidic species, and their simultaneous elimination was required for significant reduction of acidic variants. Consequently, by modulating temperature and pH to reduce PTMs and supplementing with L-cysteine to displace GSH, the proportion of acidic variants was successfully reduced. This study uncovers dual formation mechanisms of acidic species in THIOMABs and provides a practical cell culture approach, effectively reducing acidic variants and enhancing the quality attributes of THIOMABs.

## 1 Introduction

Antibody-drug conjugates (ADCs), a groundbreaking class of anticancer therapeutics, have shown immense promise in revolutionizing cancer treatment (Abdollahpour-Alitappeh et al., 2019; Beck et al., 2017; Junutula et al., 2008). They take advantage of the specificity of a monoclonal antibody to deliver a linked cytotoxic agent directly into a tumor cell (Sievers and Senter, 2013), thereby reducing toxicity to normal tissue. The cysteine-engineered antibodies, also known as THIOMABs, incorporates cysteine (Cys) at specific sites to facilitate thiol conjugation and it serves as an intermediate in the creation of more homogeneously loaded ADCs that are suitable for clinical development (Junutula et al., 2008; Junutula et al., 2010).

Antibody charge heterogeneity refers to the uneven distribution of charges caused by differences in the surface charges of antibodies. Based on their net charge, they can be broadly classified into acidic variants, main species, and basic variants. When analyzed by chromatography-based methods, acidic species and basic species are defined based on their retention times relative to the main peak. For instance, in cation exchange chromatography (CEX), the acidic variants elute prior to the main peak, while the basic variants are eluted subsequent to it (Chen et al., 2019; Neill et al., 2015; Talebi et al., 2013). The charge heterogeneity has a significant impact on both the efficacy and safety of the antibodies, while the acidic variants grabbed more attention than the basic variants due to the higher chance to impact the efficacy and safety (Boswell et al., 2010; Chung et al., 2018; Dakshinamurthy et al., 2017). Therefore, it is essential to focus on reducing the formation of acidic charge isomers during the process development stage. For traditional antibody molecules, acidic variants are primarily caused by post-translational modifications (PTMs) during antibody production, including deamidation, glycosylations, oxidation of sidechains, cysteinylation fragmentation and various other factors (Banks et al., 2008; Harris et al., 2001; Yan et al., 2009). For cysteine-engineered antibody, the charge heterogeneity becomes more complicated due to the reactive thiol group on the engineered cysteine (Landino et al., 2004; Mawatari and Murakami, 2004). This thiol group readily interacts with acidic substances in the cell culture medium, such as glutathione (GSH), altering the antibody’s surface charge distribution and elevating the proportion of acidic variants (Dörmann et al., 1993). Additionally, in charge heterogeneity analyses using CEX, this results in the transformation of a single expected peak splitting into multiple dispersed peaks, complicating detection and analysis of the engineered cysteine sites (Chen et al., 2009).

Although these acidic modifications introduced at the engineered site are generally removed during the reduction process of antibody-drug conjugation, and do not directly affect the properties of final ADCs products (Junutula et al., 2008), their presence exerts a substantial impact on the quality attributes of the antibody inter-mediate in the ADCs production. Specifically, these acidic variants compromise the intermediate’s purity and stability, posing challenges to subsequent manufacturing and quality control processes. In addition, they hinder the development of charge-based assays, such as CEX analysis using high performance liquid chromatography (CEX-HPLC) and imaged capillary isoelectric focusing (iCIEF) for accurate characterization of the antibody intermediate (Chen et al., 2009). Since this acidic peak is generated during the antibody expression process, gaining a thorough understanding of its production process becomes particularly crucial. THOMABs are mainly produced in Chinese hamster ovary (CHO) cells, which are the most utilized platform for the production of their formation mechanism during CHO cell culture is necessary. By meticulously examining the correlation between cell culture conditions and acidic charge variants, the key factors influencing their formation can be identified. This identification allows for the effective reduction of acidic charge variant generation through precise control of culture conditions during the cell culture process.

In this study, we initially demonstrated that the addition of L-cysteine effectively competes with GSH to mitigate GSH capping, although it did not alter the levels of acidic variants. Subsequently, by modulating temperature and pH to suppress PTMs such as deamidation, we observed a slight reduction in the content of acidic variants. When these two strategies were applied in combination, the proportion of acidic species was dramatically reduced from 63.57% to 32.12%. This represents the first report elucidating the dual impact of GSH capping and traditional PTMs on the formation of acidic isomers in THIOMABs, offering both theoretical insights and practical strategies to optimize cell culture processes for THIOMAB in ADC production.

## 2 Materials and methods

### 2.1 Cell lines

The cell line used in this study is AmigoCHO, a proprietary cell line developed by AsymBio, it was constructed using the host cell line CHO-K1, originally sourced from ECACC (Lot: 16H036). The host cells were stably transfected with an expression vector (pAS5.0 with CMV promoter and SV40 poly(A) signal, synthesized by Genewiz, Suzhou, China) containing the target gene and a glutamine synthetase (GS) selection marker. The resulting engineered molecule features four strategically incorporated engineered cysteines. These transfected cells were subsequently cultured in CD02 medium (QuaCell, Shanghai, China) supplemented with methionine sulphoximine (MSX) (Sigma-Aldrich, Darmstadt, Germany) to enable stable expression and secretion of IgG1-based THIOMAB variants.

### 2.2 Cell thawing and expansion

For cell thawing, frozen cells were incubated at 37 °C until complete thawing was achieved. Following, the content of the vial was gently transferred to an appropriate amount of pre-warmed medium to achieve an inoculation cell density of (0.3–0.5) × 10^6^ cells/mL. The cells were cultivated in shake flask in a humidified atmosphere at 36.5 °C and 6% CO_2_ under orbital shaking with 120 revolutions per minute (rpm). The cells were sub-cultivated every 2–3 days and inoculation was performed by a simple split into CD02. Cells were passaged and maintained under MSX selective pressure until production culture.

### 2.3 Fed-batch procedure

For fed-batch procedures, production medium Actipro (HyClone, Cytiva, SG) and feed media Cell Boost 7a (CB7a) (HyClone, Cytiva, SG) and Cell Boost 7b (CB7b) (HyClone, Cytiva, SG) were used. The feeding volume ratio of Cell Boost 7a to 7b is 10:1 (v/v). On day 3, day 5, day 7, day 9, and day 11, the feed volumes for Cell Boost 7a and 7b were set at 4% and 0.4% of the initial culture volume, respectively. On day 12, the feed volumes were adjusted to 2.0% and 0.2% for Cell Boost 7a and 7b, the performed strategy can be adjusted based on cell growth and metabolism. The inoculation density was (0.4–0.6) × 10^6^ cells/mL for spin tubes or bioreactors. For spin tubes, the cells were cultivated at 36.5 °C (shifted to 33 °C when the cell density was (12–16) × 10^6^ cells/mL, 6% CO_2_ and 80% humidity under orbital shaking with 225 rpm. Cells were cultivated for up to 14 days or if viability dropped below 70% and fed with media CB7a and CB7b as well as glucose according to a standard feeding regimen. Viable cell density (VCD), viabilities, product titer as well as osmolality, glucose and lactate concentrations were determined at different time points throughout the process.

### 2.4 Bioreactor operations

Bioreactor runs were performed in 3 L bioreactors equipped with a pH probe, DO probe, a pitched 3-blade impeller, a ring sparger system for gas supply, and feeding tubes for the supplement of CB7a, CB7b, antifoam solution, glucose solution or additive according to the experimental design. The following set points and process ranges were maintained: temperature (temp) 36.5 °C (shifted to 33.0 °C or 32 °C when the cell density was (12–16) × 10^6^ cells/mL, pH 7.00 ± 0.20 (not adjusted or adjusted to 6.90 ± 0.15 after temp shifted), dissolved oxygen 40%, fixed air sparging rate of 0.0067 vvm (air volume/culture volume/min). The dissolved oxygen level was controlled by a PID control loop. The pH was maintained using sodium carbonate addition and carbon dioxide sparging. Stirrer speed was stirred at 250 rpm.

### 2.5 Study design

The cell culture process was developed using a standard fed-batch approach in spin tubes and 3 L bench-top bioreators. This study consisted of two rounds of experiments. In the first round, six experimental groups were conducted in spin tubes for additive screening, including one control group and four experimental groups aimed at reducing GSH capping, as well as one group to verify GSH capping at the engineered sites. L-Cys (Aladdin Scientific, Shanghai, China) and L-cystine ((Aladdin Scientific, Shanghai, China) were each added at 5 mM on days 5, 8, and 11, ZnSO_4_ (Aladdin Scientific, Shanghai, China) was introduced at 40 μM on day 3, methionine ((Aladdin Scientific, Shanghai, China) was incorporated at 5 mM into the basic medium, and GSH (Aladdin Scientific, Shanghai, China) was added at 2 mM on days 5, 8, and 11. In the second round, three experimental groups were designed in 3 L bioreactors: a blank control group, a group utilizing the low temp and low pH control strategy, and a group combining the low temp and low pH strategy with the additive L-Cys. The L-Cys was added at 5 mM on days D5, D8, and D11, respectively. Specifically, the purpose and key factors are detailed in Table 1.

**TABLE 1:** The purpose and key factors in the study.

Run	Purpose	Working volume & culture vessels	Variables
1st round of study	Additives screening to displace GSH	20 mL in 50 mL spin tubes	Control
			L-Cysteine
			L-Cystine
			ZnSO_4_
			Methionine
			GSH
2nd round of study	Bioreactor conditions to reduce PTMs	1.5 L in 3 L bioreactors	Control (Temp adjusted to 33.0 °C; pH maintained at 7.00 ± 0.20)
			Low temp & low pH (Temp adjusted to 32.0 °C; pH adjusted to 6.90 ± 0.15)
	Combined strategy to minimize acidic species		Combination (Temp adjusted to 32.0 °C; pH adjusted to 6.90 ± 0.15; L-Cysteine)

### 2.6 Protein purification

Antibody protein purification (one-step affinity chromatography) was performed through a semi-automated protein purification system AmMag^TM^SA Plus (GenScript, Nanjing, China). The harvested sample was centrifuged to collect the supernatant, based on binding capacity calculations, an appropriate volume of supernatant was added to PBS-equilibrated magnetic beads (1 mL beads with 60 mg protein binding capacity). The mixture was sealed and incubated on a thermostatic shaker at room temperature for 2 h. Purification was performed using the AmMag^TM^SA Plus, with the following program: 3 × washes with PBS, 1 × wash with H_2_O, and 1 × elution with 0.1 M NaAC-HAC buffer (pH 3.0). After elution, the concentration of the sample ranged between 10 and 20 mg/mL, and the pH was adjusted to 5.5 using tris-base (pH 11.0).

### 2.7 Online measurements and offline analysis

The cell number and viability were determined using an automated cell counter (Countstar, ALIT, China). Blood gas analysis (BGA) was performed with a blood gas analyzer (ABL9, Denmark), measuring parameters including pH, pCO_2_, and pO_2_. Osmolality was assessed using an osmolality meter (OSMO-MAT AUTO, Germany), with a sample volume of 50 μL (centrifuged supernatant). Glucose and lactate levels were quantified by a glucose and acid analyzer (TANKBIO C-line 20, Germany), utilizing a 10 μL sample-to-reagent ratio of 1:50. pH and DO were monitored using pH/DO sensors (Hamilton, Switzerland), with real-time online monitoring of reactor pH/DO parameters, daily offline BGA sampling was conducted, and online values were calibrated to offline values when pH deviations ≥0.05 were observed. Charge heterogeneity and titer were analyzed via high-performance liquid chromatography (Agilent 1,260, United States) using a POROS™ 20A column (4.6 mm × 50 mm, Thermo Scientific™). The mobile phase consisted of phosphate buffers: Phase A (0.2 M Na_2_HPO_4_, pH 6.75 ± 0.05) and Phase B (0.2 M NaH_2_PO_4_, pH 3.0 ± 0.05), with a flow rate of 1.0 mL/min under gradient elution. Detection was performed at 280 nm.

### 2.8 Deglycosylated intact mass (DIM) analysis

Deglycosylated intact mass was determined using a high-resolution liquid chromatography-mass spectrometry (LC-MS) system (Waters Xevo G2-XS QTof, United States). For sample preparation, 50 μg of protein was diluted to 50 μL with 0.1 M Tris-HCl (pH 7.5), and then 1 μL Rapid PNGase F (enzymatic deglycosylation) was added. The mixture was incubated at 50 °C with shaking (500 rpm) for 30 min. The reaction was terminated with 1 μL 10% formic acid, and centrifuged at 13,000rcf for 5 min. The supernatant (40–45 μL) was transferred to a liquid chromatography vial. The sample was separated on a Bioresolve RP mAb Poly column (2.1 × 100 mm, 2.7 μm; Waters) at 80 °C, using a mobile phase gradient of 5–90% B (0.1% formic acid in acetonitrile) over 12 min at 0.4 mL/min. Mass spectrometry was conducted in positive ion mode with a capillary voltage of 3 kV.

### 2.9 Post - Translational modification (PTM) analysis

Protein samples are concentrated using a 10 kDa molecular weight cut - off (MWCO) ultrafiltration tube with 0.1 M Tris - HCl (pH 7.6 ± 0.05) until the sample concentration exceeds 5 mg/mL. Then, the samples are denatured with 8 M Gdn·HCl - 0.1 M Tris - HCl and reduced with 1.0 M dithiothreitol (DTT) at 37 °C for 30 min. Cysteine alkylation is carried out using 0.5 M iodoacetamide (20 μL) at room temperature for 30 min in the dark. Buffer exchange is performed through a three-step centrifugation process (6 M urea - Tris - HCl → Tris - HCl) to remove excess reagents. The recovered protein is digested with Lys - C (enzyme-to-protein ratio of 1:20) at 37 °C for 4 h, and the reaction is terminated with 10% formic acid. Peptides are separated by high - performance liquid chromatography using a BEH C18 column with a gradient of 0.1% formic acid in water (A) and acetonitrile (B). Data-dependent acquisition is performed in positive ion mode electrospray ionization - tandem mass spectrometry (ESI - MS/MS) on QE Plus. Raw data are processed by Byos.

### 2.10 CEX peak classification analysis

Cation-exchange chromatography (CEX) was carried out on a high-performance liquid chromatography Agilent 1,260 infinity Ⅱ (Agilent Inc., Santa Clara, CA, United States) equipped with a BioPro IEX SF 4.6 mm × 100 mm column (YMC Co., Ltd., Kyoto, Japan). Forty 40 μg of mAb was injected onto the column equilibrated in buffer A (20 mM PB, with the pH adjusted to 6.00) and held for 5 min at 0% buffer B (100 mM sodium chloride in buffer A). The antibody was eluted by a linear gradient to 80% buffer B over a 30-min period with detection at 280 nm. The column temperature and flow rate were maintained at 45 °C and 0.8 mL/min, respectively. The samples were reasonably integrated using Empower software. Each peak was assigned to a Timed Group according to the typical peak attribution method in chromatograms. The proportion of each component peak was automatically calculated by Empower and reported as the final result.

In this study, based on the elution order of charge variants in CEX, peaks 1, 2 and 3 are categorized as acidic peaks, peak 4 as the main peak, and peaks 5, 6 and 7 as basic peaks (Supplementary Figure S1).

## 3 Results

### 3.1 The effect of diverse additives on acidic variants

THIOMAB is an engineered antibody featuring a cysteine residue where its free thiol group is normally capped by forming a disulfide bond with another cysteine. During cell culture, however, this free thiol group may bind to other thiol-containing acidic substances in the fermentation broth, leading to an increase in the number of acidic groups and subsequently elevated acidic peaks. Furthermore, the introduction of acidic charges may interfere with the overall antibody, altering its charge properties. Based on the investigation of capping forms at the engineered site (Chen et al., 2009; Procopio-Melino et al., 2022; Zhong et al., 2017), we have deduced that this acidic substance is glutathione (GSH), which contains a thiol group. In order to minimize GSH capping at the engineered site, the experimental design involves the utilization of additives with diverse mechanisms of action, such as increasing the competitive capping ratio of Cys at engineered sites, accelerating the TCA cycle to promote GSH metabolism, sulfur-containing amino acids, oxidation, altering the surface charge of antibodies (Supplementary Table S1). This study was conducted in 50 mL shaking tubes, with an initial culture volume of 20 mL in each tube. The cultivation process followed the protocols outlined in sections 2.3, 2.4 and 2.6.

**FIGURE 1:**
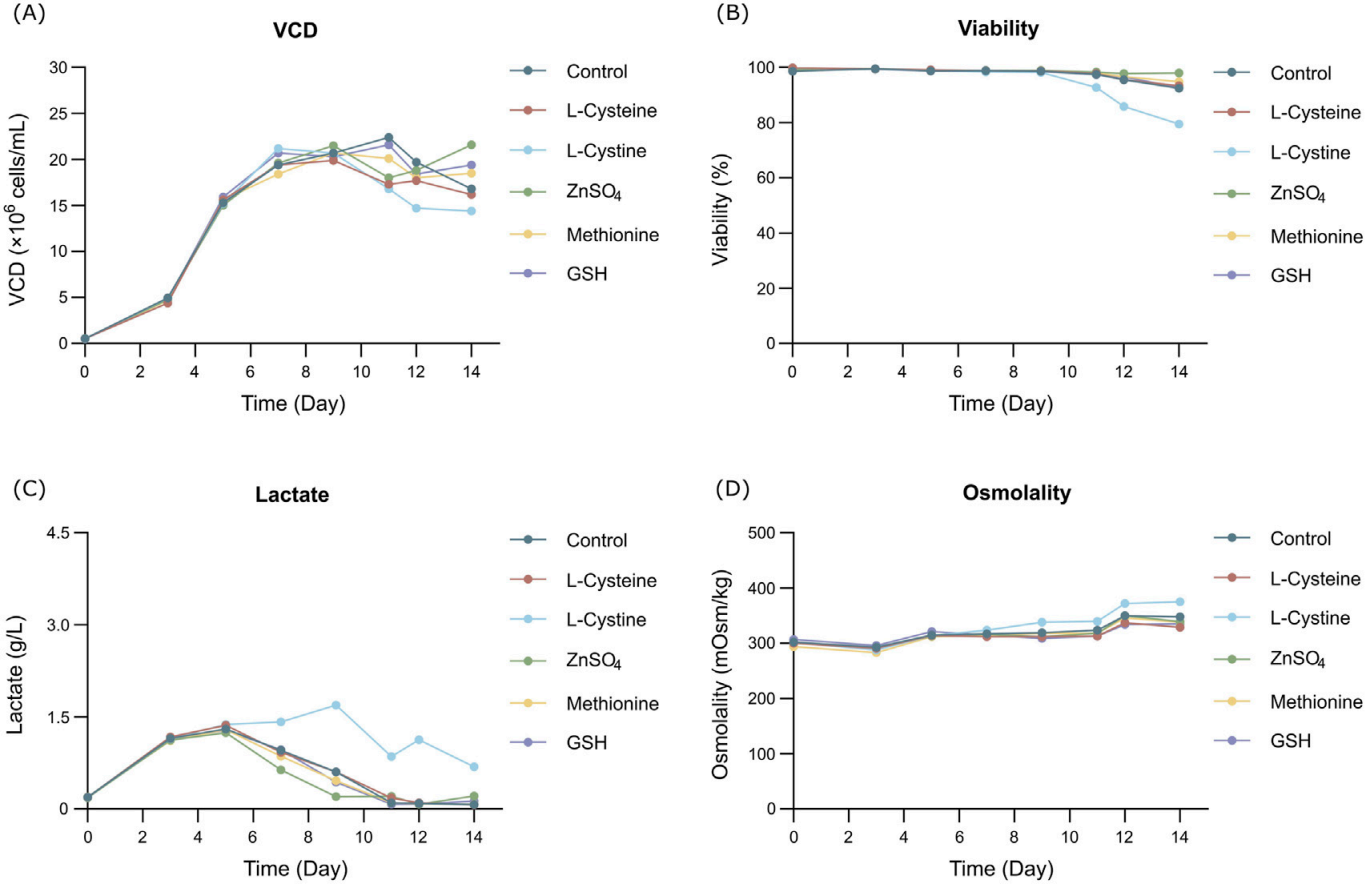
Results of fed-batch CHO cell culture processes in the additives screening study. **(A)** Viable cell density. **(B)** Cell viability. **(C)** Lactate concentration. **(D)** Osmolality.

The cell growth and metabolism results showed the cell growth curves were comparable between different additives with peak VCDs around 19.5 × 10^6^ cells/mL (Figure 1A) and viability above 90% (Figure 1B) at harvest day 14, the cell viability of the L-cystine group was 79.43%, which can be explained by the excessively high pH of the alkali-soluble L-cystine added in the later stage. Except the group of L-cystine, the trend for lactate proportion was the same, it accumulated rapidly in the early stage of cell culture, and then decreased gradually to near zero and maintained until the harvest (Figure 1C). The osmolality of the different groups ranged from 280 to 380 mOsm/kg during the whole cell culture period (Figure 1D). With the exception of the L-cystine group, all experimental groups demonstrated productivity levels comparable to the control group, yielding approximately 6.24 g/L at harvest (Table 3). Quality analysis results performed by DIM demonstrated that the engineered site indeed contains GSH, the antibody’s four engineered cysteines caused diverse capping patterns due to varying GSH/Cys binding amounts (Table 2), further validating our hypothesis. Additionally, compared to the control group, the competitive capping effect of different forms of Cys at engineered sites can effectively enhance the proportion of Cys-capping. The addition of GSH, as the negative control, enables the increase of the GSH capping percentage at the engineered site (Table 2). However, in the CEX analysis, additives that interacted competitively with GSH or involved in its transformation did not decrease the percentage of the acidic peak. Conversely, the addition of GSH itself resulted in an increase in the percentage of the acidic peak (Table 3).

**TABLE 2:** DIM analysis in the additives screening study.

			DIM (%)				
Variable	No.	Additive	Cys (1), GSH(3)	Cys (2), GSH(2)	Cys (3), GSH(1)	Cys (4)	GSH(4)
Additive	1	Control	27.3	45.2	27.6	—	—
	2	L-Cysteine	—	—	42.5	57.5	—
	3	L-Cystine	—	—	44.3	55.7	—
	4	ZnSO_4_	24.2	44.6	31.1	—	—
	5	Methionine	22.3	44.4	33.4	—	—
	6	GSH	35.1	38.2	14.9	—	11.9

\ means not detected in this article.

**TABLE 3:** The productivity and peak proportion in the additives screening study.

				CEX (%)							
Variable	No.	Additive	Titer (g/L)	Acidic peak			Main peak	Basic peak			Acidic peaks
				Peak1	Peak2	Peak3	Peak4	Peak5	Peak6	Peak7	
Additive	1	Control	6.24	31.67	20.29	13.20	16.86	4.60	5.99	7.40	65.16
	2	L-Cysteine	6.26	37.45	21.29	4.73	26.79	4.62	2.01	3.11	63.47
	3	L-Cystine	5.42	48.24	18.30	4.62	19.39	3.71	2.24	3.50	71.16
	4	ZnSO_4_	6.07	29.73	19.50	13.34	18.38	4.83	6.89	7.33	62.57
	5	Methionine	6.43	27.95	19.64	12.88	21.82	2.86	7.67	7.19	60.47
	6	GSH	6.51	44.66	19.15	13.62	10.81	3.67	3.31	4.77	77.43

The results showed that the addition of L-Cys could reduce GSH at the engineered site, but the proportion of acidic peaks did not decrease. By contrast, the addition of GSH could increase the proportion of GSH and also elevate the ratio of acidic peaks. This findings suggest GSH capping at the engineered site contributes to the formation of acidic peaks. More importantly, the modifications in Cys capping alone are not the sole critical factor contributing to the formation of acidic peaks. Instead, the generation of acidic variants appears to be influenced by additional underlying mechanisms.

### 3.2 The effect of diverse bioreactor parameters on acidic variants

THIOMAB is essentially an antibody, and the antibody itself also have acidic charge isomers, mainly due to traditional PTMs such as deamidation, oxidation, glycosylation, etc. Among them, studies have shown that deamidation is considered to be the most common contributor to acidic charge isomers and accounts for the largest proportion (Deamidation of Proteins, 2019; Gupta et al., 2022). To independently investigate the impact of traditional PTMs on the acidic peak of the THIOMAB, a strategy combing low pH and low temp, previously validated effective for PTMs management in AsymBio cell culture platform, was employed in this study. For all bioreactors, the initial culture volume was set at 1.5 L, the initial pH control process was maintained at 7.00 ± 0.20, and the initial culture temp was set at 36.5 °C. In the control group, the temp was reduced to 33 °C, with no adjustment made to the pH. In the experimental group, the temp was lowered to 32 °C, and the pH was adjusted to 6.90 ± 0.15. All other culture parameters remained consistent, and standard fed-batch procedure was conducted using 3 L bioreactors for both groups.

**FIGURE 2:**
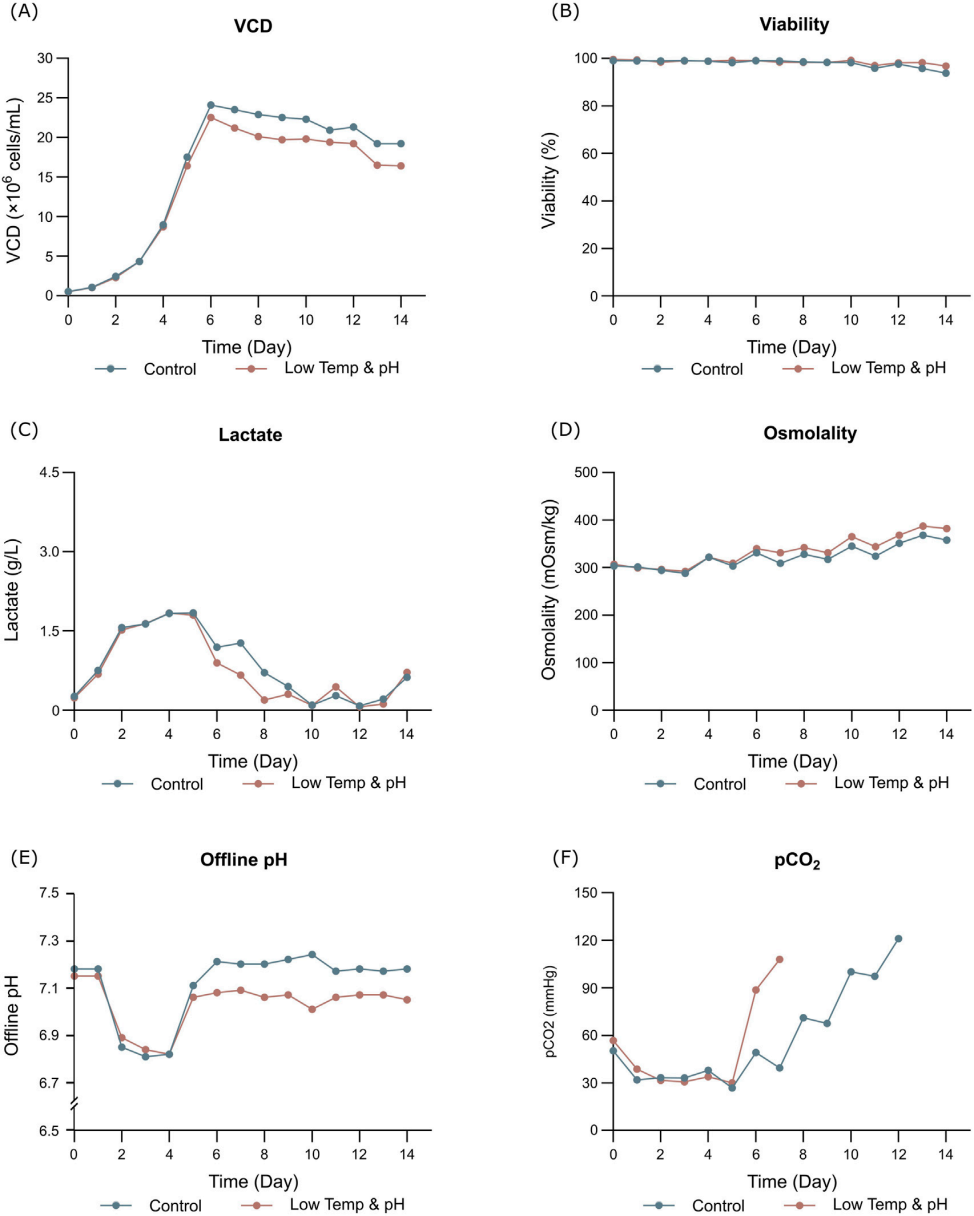
Results of fed-batch CHO cell culture processes in the bioreactor conditions study. **(A)** Viable cell density. **(B)** Cell viability. **(C)** Lactate concentration. **(D)** Osmolality. **(E)** Offline pH. **(F)** pCO_2_.

**FIGURE 3:**
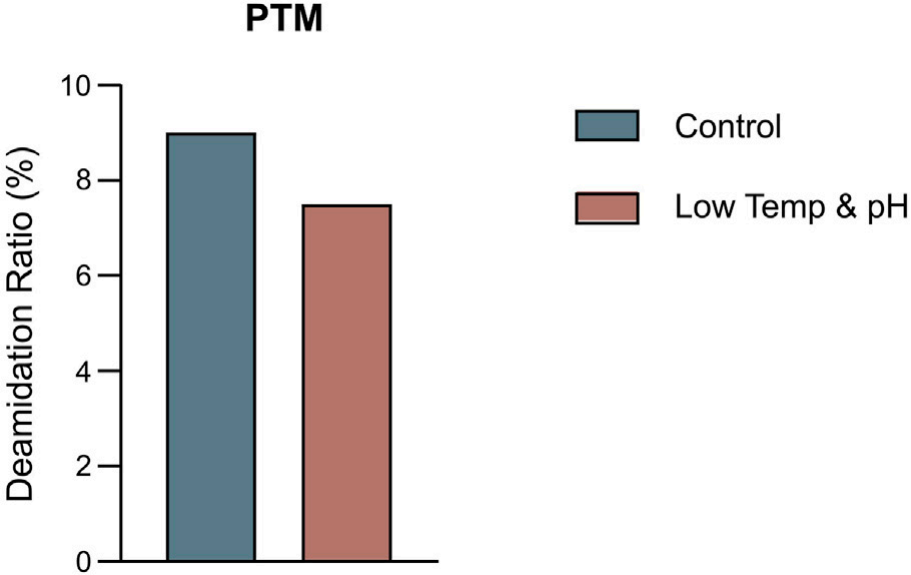
The percentage of deamidation in PTMs analysis by intact LC-MS in the bioreactor conditions study.

The results showed that the cell growth under different culture conditions were basically consistent, with peak VCDs all exceeding 20 × 10^6^ cells/mL (Figure 2A) and viability at harvest remaining above 90.0% (Figure 2B). The profiles of lactate and osmolality showed the similar trends, the osmolality and lactate ranging from 280 to 390 mOsm/kg and 0.2–1.9 g/L the throughout the whole culture period respectively (Figures 2C,D). The off-line pH decreased to about 6.8 with the increase of lactate concentration at the early stage of culture, after that, with consumption of lactate, the pH returned gradually to the upper limit (7.05 for the low pH group, and 7.2 for the control) (Figure 2E). Additional CO_2_ was required in the low pH group at the late stage of culture to maintain low pH, therefore, pCO_2_ went up continuously until reaching the pCO_2_ detection limit of the equipment (Figure 2F). The titer declined slightly, with the control group reaching 6.53 g/L and the low temp and low pH group achieving 5.14 g/L (Table 4). CEX analysis confirmed that acidic variants were reduced under lower temp and pH conditions, with their proportion decreasing from 63.57% to 50.13% (Table 4), and the detailed CEX profiles are presented in Supplementary Material (Figures 2A,B). To determine whether this reduction was driven by PTM changes, a PTM analysis was conducted (Supplementary Table S3). Deamidation and Pyro-Q formation which are reported as the primary contributor to the generation of acidic peaks (Du et al., 2014) are detected in this PTM analysis. Under low pH and low-temperature conditions, the proportion of Pyro-Q formation remained unchanged, while deamidation levels decreased by 16.7% compared to the control group (Figure 3; Supplementary Table S3). This indicates that the reduction in acidic variants was solely attributed to the decrease in deamidation. Other detected modifications, including succinimide formation, methionine oxidation, Lys-loss, and amidation (based on Lys-loss and Gly-loss) primarily influenced the change of basic isomers (Chen et al., 2009; Du et al., 2014; An et al., 2014). Additionally, DIM analysis revealed a high proportion of GSH capping in both groups (Table 5), and the detailed DIM profiles are presented in Supplementary Material (Figures 3A,B).

**TABLE 4:** The productivity and peak proportion in the bioreactor conditions study.

			CEX (%)								
Variable	Group	Titer (g/L)	Acidic peak			Main peak	Basic peak				Acidic peaks
			Peak1	Peak2	Peak3	Peak4	Peak5	Peak6	Peak7		
Temp & pH	Control	6.53	31.38	19.24	12.95	16.51	4.64	5.85		9.42	63.57
	Low Temp & pH	5.14	19.46	15.49	15.18	20.59	8.55	10.83		9.91	50.13

**TABLE 5:** DIM analysis in the bioreactor conditions study.

		DIM (%)				
Variable	Group	Cys (1), GSH(3)	Cys (2), GSH(2)	Cys (3), GSH(1)	Cys (4)	GSH(4)
Temp & pH	Control	22.5	42.6	27.0	7.9	—
	Low Temp & pH	16.7	36.8	33.3	13.2	—

The findings indicated that low temp and low pH conditions effectively mitigated PTMs, yet the acidic peaks demonstrated only a marginal decline, which fell short of our anticipated results. Considering the contribution of GSH modification at engineered sites as explored in Section 3.1, an inference can be drawn that there is a possible overlapping effect between GSH capping at engineered sites and traditional PTMs on the formation of acidic peaks. In other words, eliminating only one of these factors may not be sufficient to completely diminish their influence on acidic peaks, as the other factor may still play a role.

### 3.3 The combined effect of additives and bioreactor parameters on acidic variants

According to the previous findings, the acidic peak of THIOMABs is possible to be influenced by the combined effects of GSH capping and traditional PTMs. Therefore, we employed a combined strategy to regulate the acidic varaints. Specifically, this involved utilizing the additive L-Cys to decrease GSH capping at the engineered sites and implementing low temp and low pH control in the bioreactor to mitigate traditional PTMs. The parameters of the bioreactor remained the same as those described in section 3.2, and the additive of L-Cys was added at a concentration of 5 mM on days 5, 8, and 11 respectively.

**FIGURE 4:**
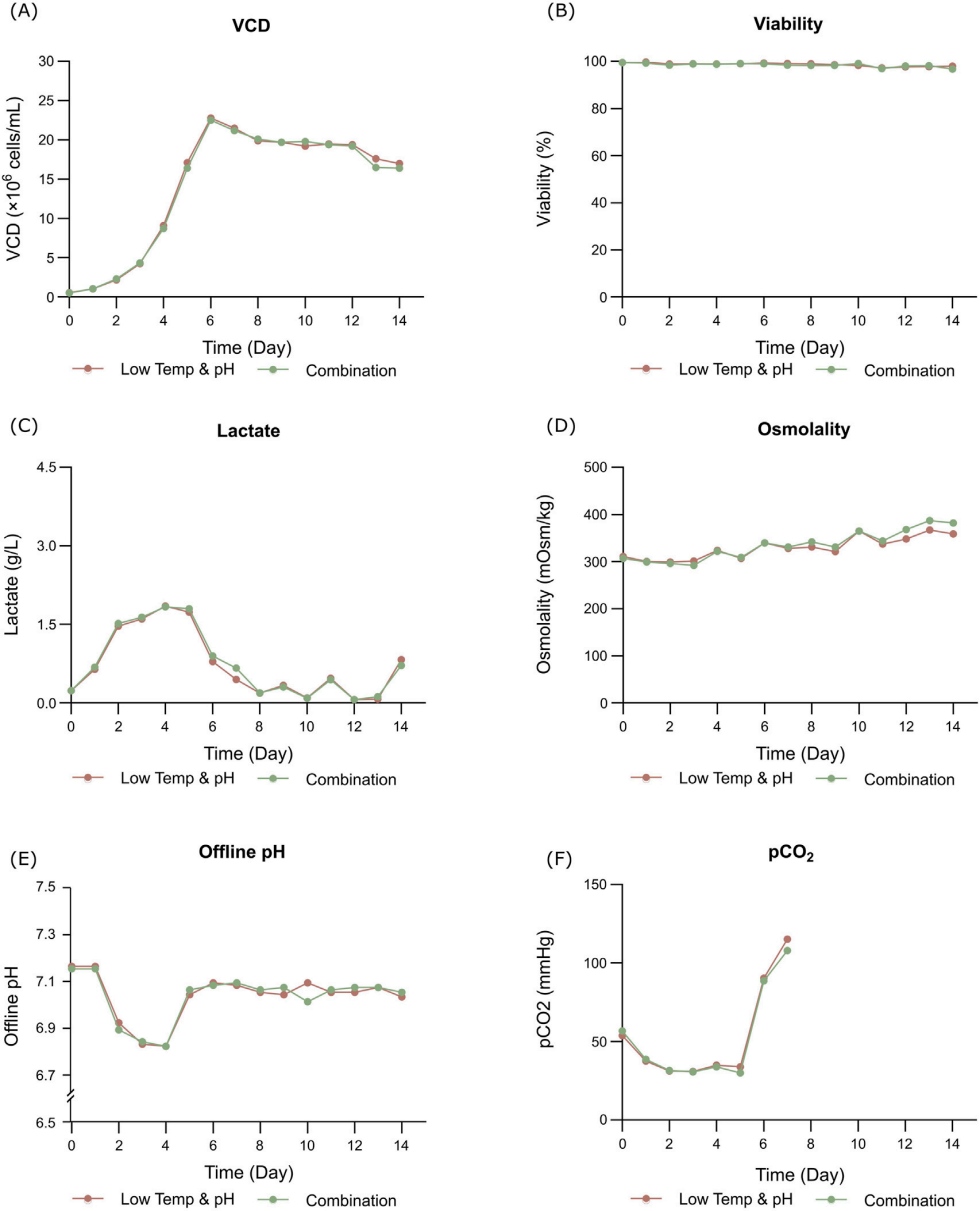
Results of fed-batch CHO cell culture processes in the bioreactor condition and combined strategy study. **(A)** Viable cell density. **(B)** Cell viability. **(C)** Lactate concentration. **(D)** Osmolality. **(E)** Offline pH. **(F)** pCO_2_.

**FIGURE 5:**
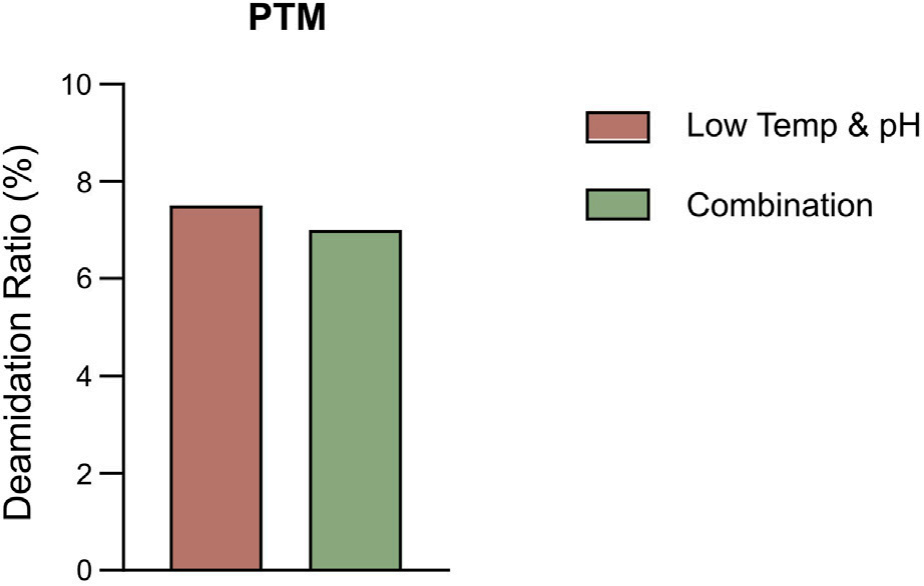
The percentage of deamidation in PTMs analysis by intact LC-MS in the bioreactor condition and combined strategy study.

The bioreactor exhibited comparable trends in terms of overall growth and metabolic profiles to the lower temp and pH condition described in Section 3.2 (Figures 4A–F), and the data on low tempe and low pH obtained from Section 3.2 is reused as a control for the combination study. The peak VCD, viability rate, lactate and osmolality at harvest time were 22.8 × 10^6^ cells/mL, 97.95%, 0.826 g/L and 359 mOsm/kg, respectively. The off-line pH decreased to about 6.80 with the increase of lactate concentration at the early stage of culture, and then the pH returned gradually to the upper limit of 7.05 with the consumption of lactate. pCO_2_ levels went up continuously until day 7 reaching the pCO_2_ detection limit of the equipment. The addition of L-Cys had no effect on the titer compared to the low temp and low pH group described in Section 3.2, which remained constant at 5.16 g/L (Table 6). CEX analysis demonstrated that L-Cys addition reduced the acidic peak proportion from 50.13% (low temp and low pH group) to 32.12% (Table 6), and the acidic peak showed a groundbreaking 50% reduction compared to the control group, the detailed CEX profiles are presented in Supplementary Material (Figure 2C). DIM confirmed a reduction in GSH capping to 10.6%, compared to 39.3% in the group using traditional PTM regulation alone. This was accompanied by a corresponding increase in Cys capping (Table 7), and the detailed DIM profiles are presented in Supplementary Material (Figure 3C). Additionally, PTM analysis focusing on deamidation modifications (N388 and N393) indicated a 22.2% reduction (Figure 5; Supplementary Table S3). This further substantiates the dual formation mechanism of the acidic peak in THIOMAB, with a definite degree of overlap between them.

**TABLE 6:** The productivity and peak proportion in the bioreactor condition and combined strategy study.

			CEX (%)							
Variable	Group	Titer (g/L)	Acidic peak			Main peak	Basic peak			Acidic peaks
			Peak1	Peak2	Peak3	Peak4	Peak5	Peak6	Peak7	
Combination	Low Temp & pH	5.14	19.46	15.49	15.18	20.59	8.55	10.83	9.91	50.13
	Combination	5.16	15.11	6.56	10.45	20.42	7.47	26.45	13.55	32.12

The data of low temp & pH are reused as a control for the combination study.

**TABLE 7:** DIM analysis in the bioreactor condition and combined strategy study.

		DIM (%)				
Variable	Group	Cys (1), GSH(3)	Cys (2), GSH(2)	Cys (3), GSH(1)	Cys (4)	GSH(4)
Combination	Low Temp & pH	16.7	36.8	33.3	13.2	—
	Combination	—	—	42.5	57.5	—

In summary, the combined use of the additive L-Cys and the low temp and low pH regulation strategy resulted in a remarkable 50% reduction in the acidic peaks of THIOMABs. Successfully lowering them to within the acceptable range of traditional antibodies. Specifically, the GSH capping modification at engineered sites was downregulated, and traditional PTMs were reduced as well. This research illustrates the dual mechanisms and their overlapping effects on the formation of acidic variants in THIOMABs. This combined strategy enhances the quality attributes and stability of ADC antibody intermediates.

## 4 Discussion

**FIGURE 6:**
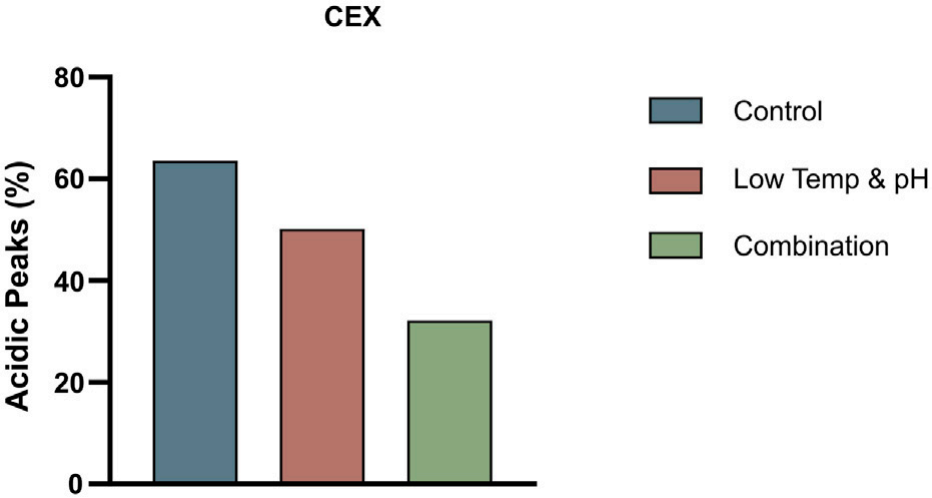
The proportion of acidic peaks in CEX under different conditions in 3 L bioreactors.

In this study, a dual mechanism for the formation of acidic species in THIOMABs was identified: GSH capping at cysteine-engineered sites and traditional PTMs during cell culture. This mechanism was found to exert overlapping effects on acidic species formation. Notably, a significant reduction in acidic variants could only be achieved through the simultaneous elimination of both influences. Specifically, the joint application of L-Cys supplementation and a low-temperature, low-pH regulation strategy resulted in a remarkable 50% reduction in acidic peaks, successfully lowering their proportion (Figure 6). This reduction aligns with the acceptable range for traditional antibodies, thereby enhancing the quality attributes and stability of the ADC intermediate. The construction methods of molecules and cell lines in this study are general, and their mechanism of action focuses on the engineered cysteine sites and the post-translational modification process undergone by the antibody itself. Therefore, this research holds typical guiding significance and broad reference value for similar THIOMAB studies.

During the cell culture, the addition of the L-Cys led to a significant replacement of GSH at engineered sites by L-Cys as revealed by DIM analysis. This finding is consistent with established research indicating a competitive replacement effect between L-Cys and GSH at engineered sites (Chen et al., 2009), nevertheless, this study directly acts on the antibody rather than involving the intracellular environment. Therefore, our study provides the first evidence from a cell culture perspective on the effectiveness of using the addition L-Cys during antibody expression. This discovery also prompted to deeply consider the role of GSH in the culture medium and how to remove GSH from the fermentation broth to avoid GSH capping issues during antibody expression. Researches have shown that GSH plays a crucial role in the growth and metabolism (such as participating in the TCA cycle and glucose metabolism, activating various enzymes, and exhibiting antioxidant and detoxifying effects) (Forman et al., 2009). However, as mentioned previously, during the process of antibody expression, GSH can undergo capping modification of the cysteine residues at the engineered THIOMAB sites, leading to an increase in the number of acidic groups and consequently causing an elevation in the acidic peak. To prevent GSH from capping engineered sites during antibody production, there are some illustrated potential solutions: 1) Screening different media to identify one that supports cell growth while minimizing GSH interference, actually, an initial culture medium screening experiment was conducted to verify the impact of different culture media on GSH component capping, and based on these screening results (Supplementary Table S4), competitive additives were introduced into the control medium for the investigative experiment presented in this study; 2) Additive substitution: Using excess L-Cys as a competitive agent to replace GSH at engineered sites; 3) Using oxidants: Altering the intracellular redox status to affect GSH levels (Zhong et al., 2017; Liu et al., 2019; Pereira et al., 2018). In this study, we adopted the strategy of direct substitution at engineered sites using additives and successfully replaced most of the GSH capping and validating our hypothesis. This finding not only provides a novel perspective on understanding the role of GSH during cell culture but also offers new strategies for optimizing antibody expression processes. Based on the section 3.1 results of this study, the proportion of acidic peaks did not decrease after GSH at engineered sites was replaced, according to the literature reports and AsymBio project experience, we further speculated that the formation of acidic charge isomers in THIOMABs is possibly related to traditional PTMs such as deamidation (Banks et al., 2008; Harris et al., 2001; Yan et al., 2009), which generates isoaspartic acid (isoAsp) or isoaspartamide (isoAsn) through the deamidation reaction of asparagine (Asn), and these products manifest as acidic variants in terms of their isoelectric points (pIs) (Sydow et al., 2014). Indeed, in this research PTM analysis confirmed the presence of deamidation (Supplementary Tables S2, S3). Subsequently, by adjusting culture parameters reduced the deamidation level of PTMs, the corresponding proportion of acidic peaks showed a slight reduction.

**FIGURE 7:**
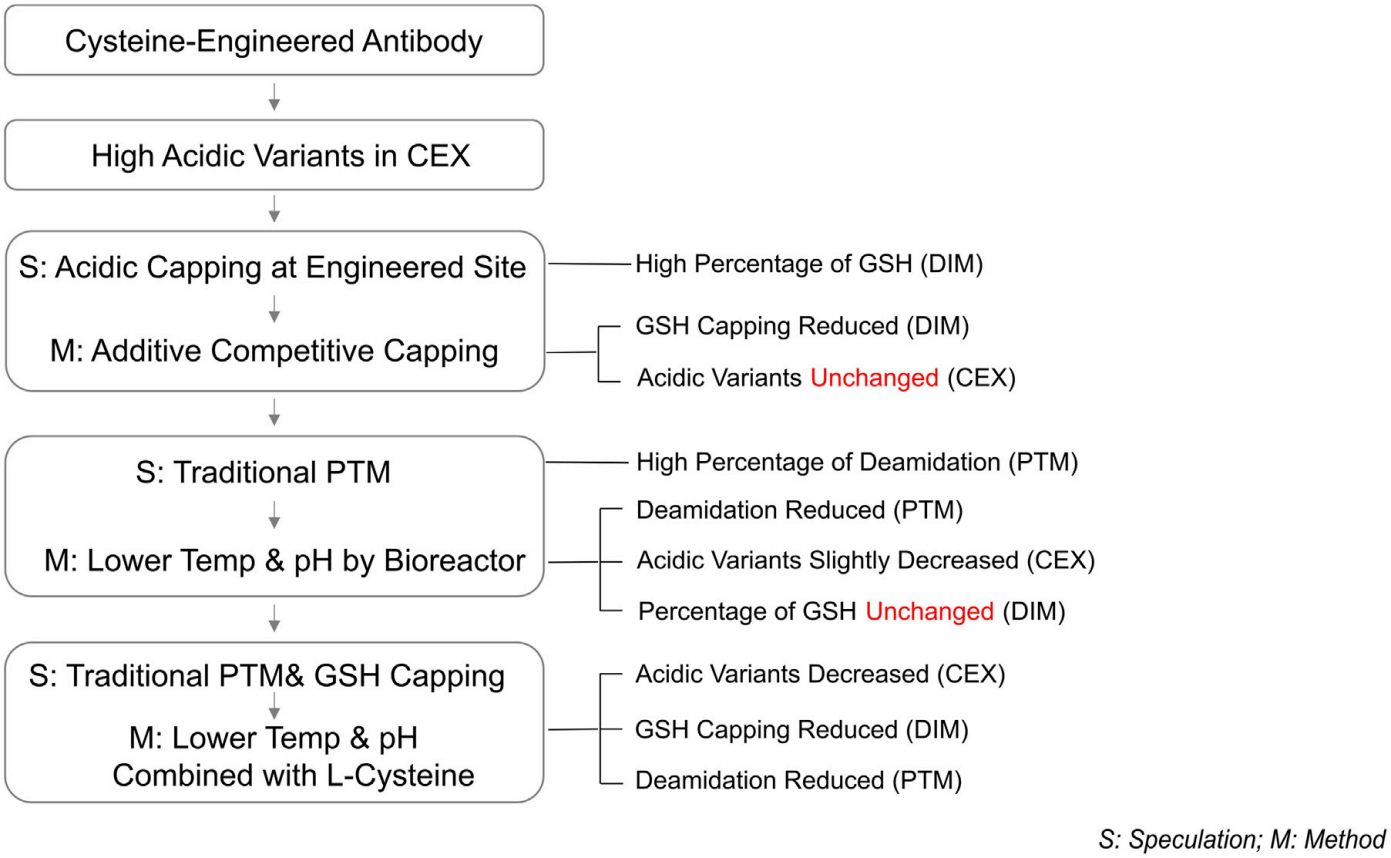
Process outline.

We observed an intriguing phenomenon: a substantial and acceptable reduction in the proportion of acidic peaks in THIOMABs occurs only when both PTMs and GSH capping at engineered sites are simultaneously minimized. This finding highlights the complexity of acidic peak formation in THIOMABs and underscores the crucial roles of these two mechanisms. Specifically, when the proportion of GSH capping modifications is reduced, the proportion of acidic peaks does not decrease significantly. This indicates that regulating the GSH capping mechanism alone is insufficient in reducing the formation of acidic peaks. Similarly, although reducing deamidation modifications in traditional PTMs lowers the acidic peak proportion, the decrease is modest. This further demonstrates that regulating a single mechanism cannot completely eliminate the influence of the other mechanism on acidic peak formation (Figure 7). Consequently, this study proposes a solution to the dual formation mechanism of acidic peaks in THIOMABs and provides a detailed explanation of the principles of action of these dual mechanisms. This discovery offers a novel perspective for understanding the formation of acidic peaks in THIOMABs. Based on this, we can further explore how to more effectively regulate these two mechanisms to maximize the reduction in the proportion of acidic peaks, providing a valuable theoretical foundation for subsequent antibody engineering and drug optimization.

## 5 Conclusion

This study successfully reduced the acidic peak ratio of THIOMABs to an acceptable level through comprehensive exploration and optimization of cell culture processes. Specifically, we introduced an innovative approach by combining the additive L-Cys with a low-temp and low-pH regulation strategy, achieving a remarkable 50% reduction in the acidic peak ratio. This advancement not only significantly improved the quality attributes of ADCs intermediates but also, for the first time, demonstrated the feasibility of reducing the proportion of acidic charge isomers in THIOMABs through cell culture strategies. This study helps lay the foundation for a deeper mechanistic understanding surrounding the formation of these acidic variants by revealing key factors which point to potential pathways that may be critically involved.

## Data Availability

The original contributions presented in the study are included in the article/Supplementary Material, further inquiries can be directed to the corresponding author.

## Abbreviations

THIOMABs, Cysteine-engineered antibodies; GSH, Glutathione; PTMs, Post-translational modifications; ADCs, Antibody-drug conjugates; CEX, Cation exchange chromatography; HPLC, High performance liquid chromatography; iCIEF, Imaged capillary isoelectric focusing; CHO, Chinese hamster ovary; MSX, Methionine sulphoximine; CB7a, Cell Boost 7a; CB7b, Cell Boost 7b; Temp, Temperature; DO, Dissolved oxygen; Vvm, Air volume/culture volume/min; LC-MS, Liquid chromatography-mass spectrometry; DIM, Deglycosylated intact mass; IsoAsp, Isoaspartic acid; IsoAsn, Isoaspartamide; Asn, Asparagine; pI, Isoelectric point; Lys, Lysine; Asp, Aspartic acid; Glu, Glutamic acid; Gly, Glycine
