# Supplementary material for: Dual formation mechanisms of acidic variants in cysteine-engineered antibodies and strategies for their reduction
Source: Front Bioeng Biotechnol. 2025 Oct 23;13:1615263. doi: 10.3389/fbioe.2025.1615263 (PMC12588982; doi:10.3389/fbioe.2025.1615263)
Supplement: Supplementary file 1 [file DataSheet1.docx]

# Supplementary Material

**Supplementary Figures and Tables**

This manuscript contains Supplementary Figure 1, Supplementary Figure 2 and Supplementary Figure 3, Supplementary Table 1, Supplementary Table 2 and Supplementary Table 3 and Supplementary Table 4.

**Supplementary Figure**

**Supplementary Figure 1.** Charge variant definitions in CEX


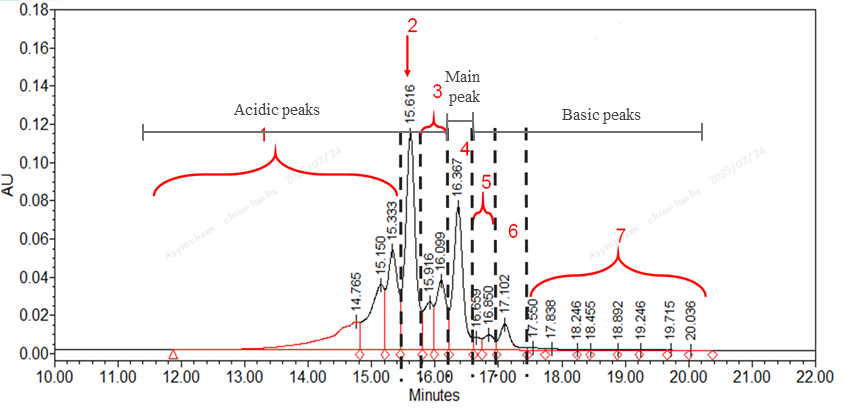


**Supplementary Figure 2** Cation exchange chromatography (CEX) profiles under different conditions in 3 L Bioreactors as shown in table 4 and table 6. (A) Control. (B) Low temp & low pH. (C) Combination.


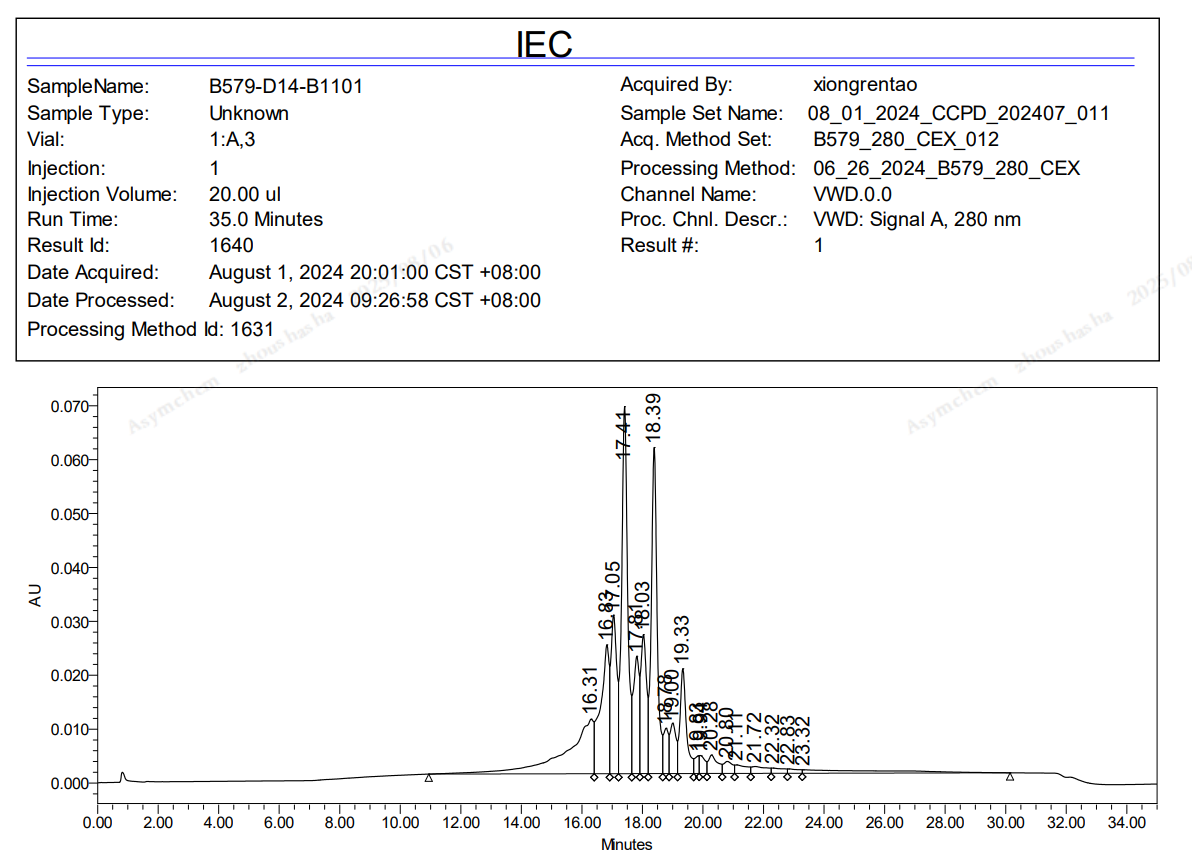


(A)


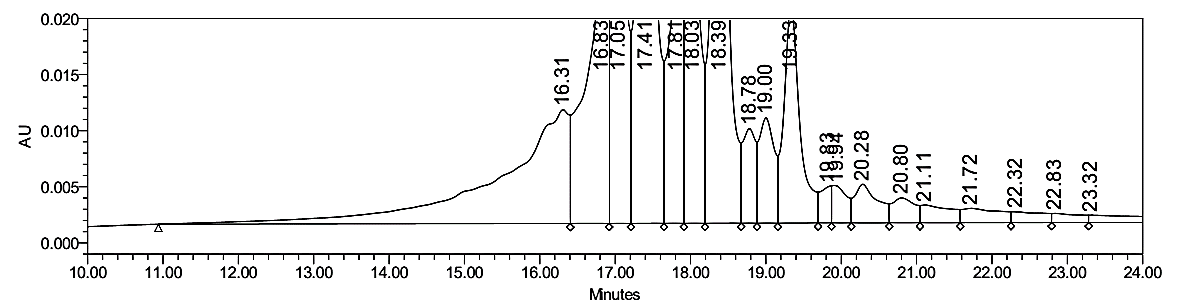

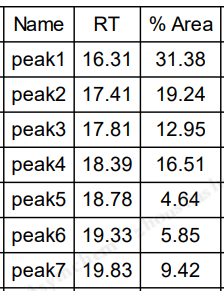

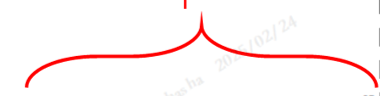


Acidic peaks

63.57%


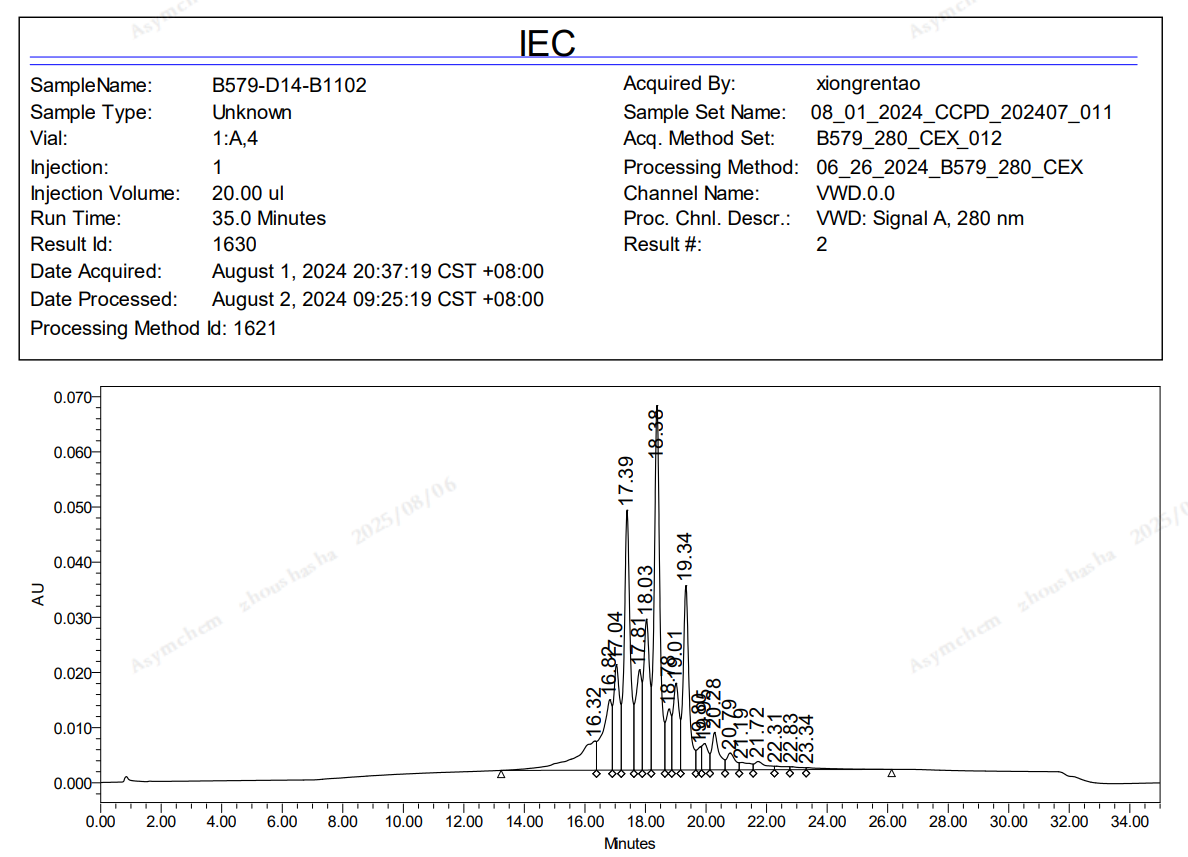


(B)


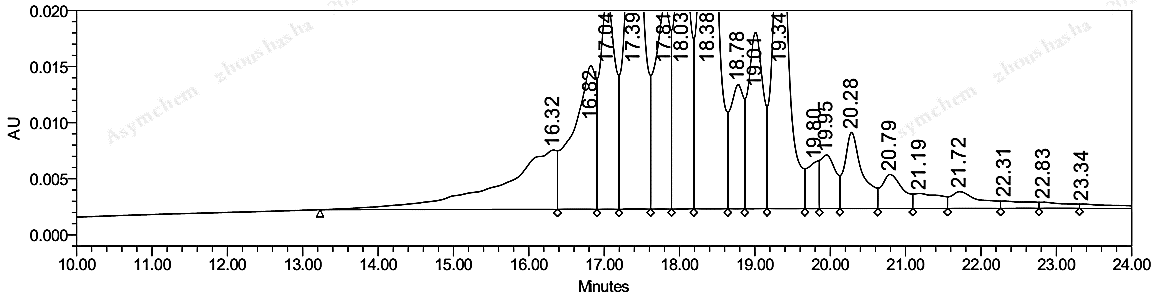

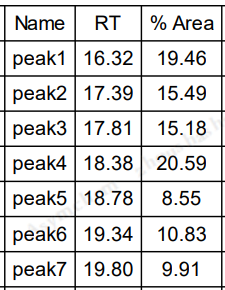

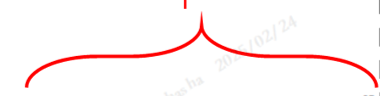


Acidic

peaks

50.13%


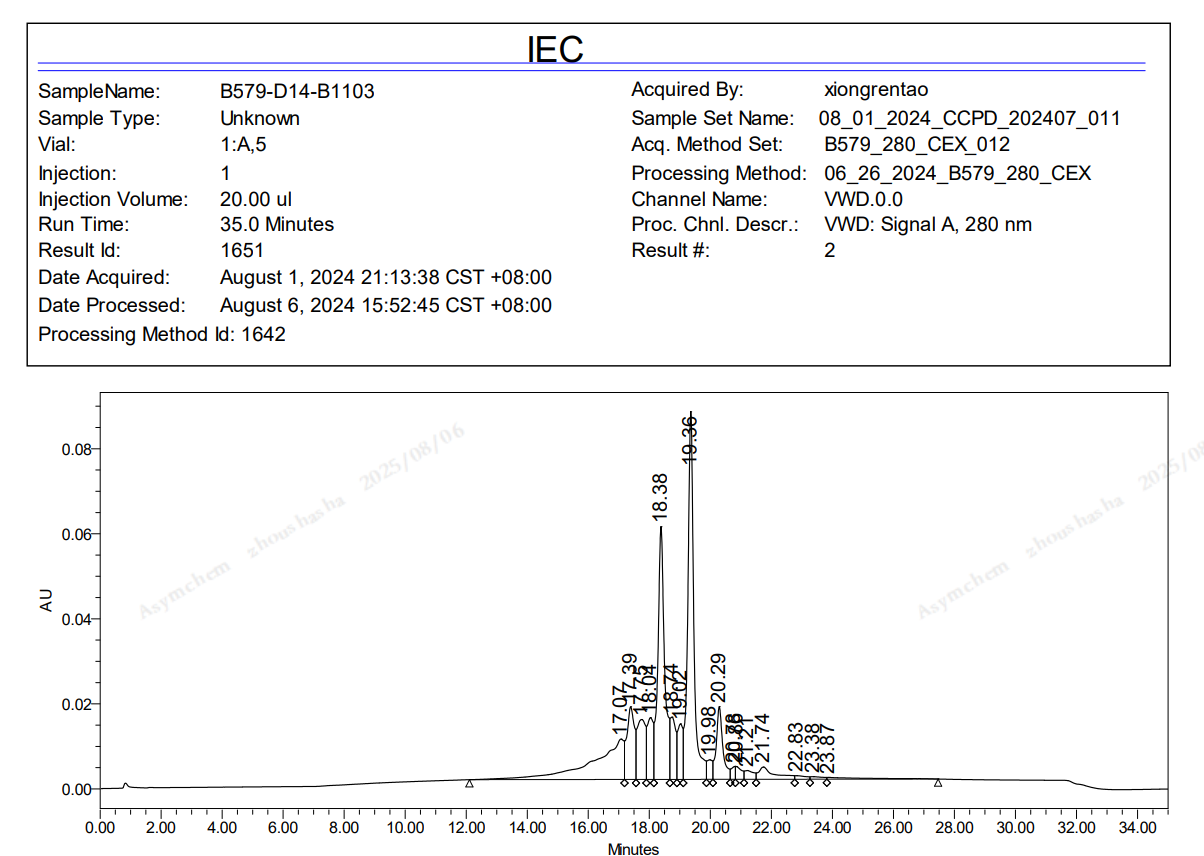


(C)


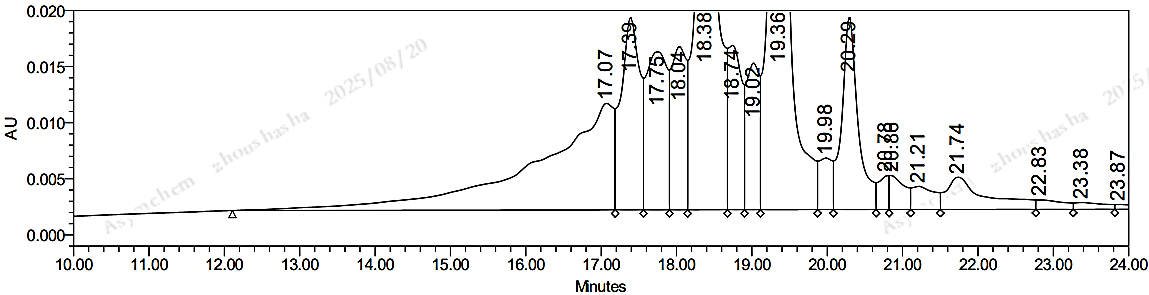

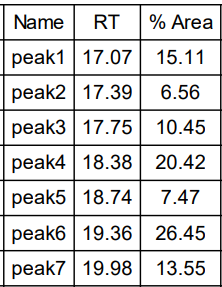

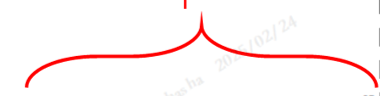


Acidic peaks

32.12%

**Supplementary Figure 3** Post-translational modification (PTM) profile under different conditions in 3 L Bioreactors as shown in table 4 and table 6. (A) Control. (B) Low temp & low pH. (C) Combination.

(A)


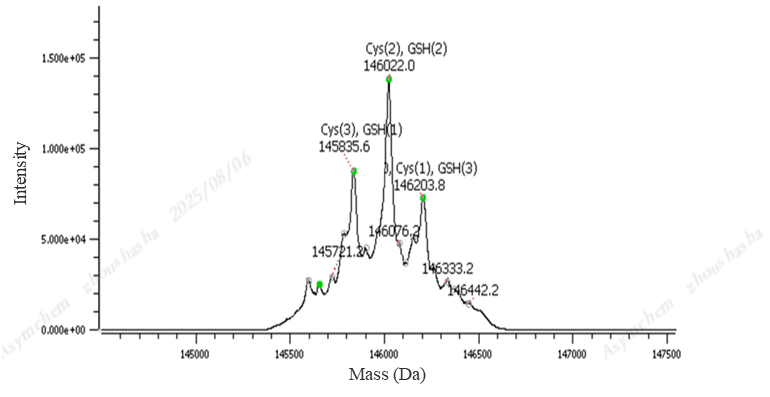


(B)


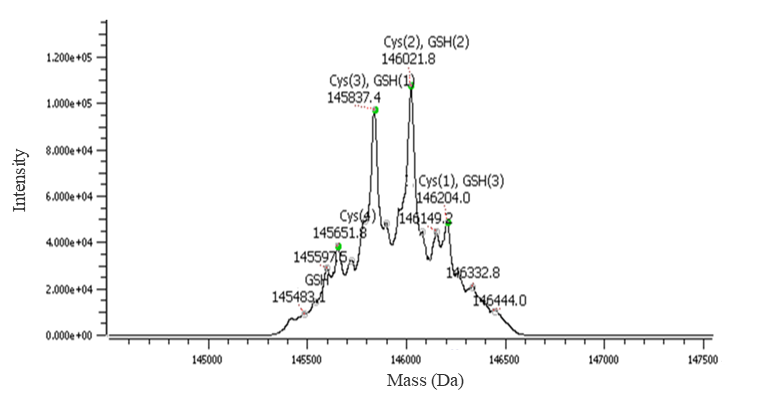


(C)


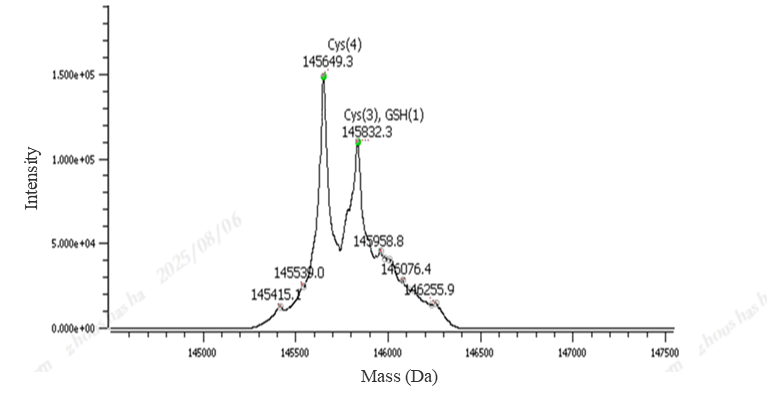


**Supplementary Tables**

**Supplementary Table 1.** Additives and their mechanisms

| Variable | No. | Group | Mechanisms |
| --- | --- | --- | --- |
| Additive | 1 | Control | Control group |
|  | 2 | L-Cysteine | Increasing the competitive capping ratio of Cys at engineered sites |
|  | 3 | L-Cystine |  |
|  | 4 | ZnSO_4_ | Accelerate the TCA cycle to promote glutathione metabolism |
|  | 5 | Methionine | Sulfur-containing amino acids, oxidation, altering the surface charge of antibodies |
|  | 6 | GSH | Perform reverse validation to investigate the impact on the capping of engineered sites |

**Supplementary Table 2.** Post-translational modifications that form acidic and basic species

| Modification types | |
| --- | --- |
| Acidic variants | Basic variants |
| Deamidation | C-terminal Lys |
| Glycation | N-terminal Glu |
| Sialylation | Isomerization of Asp |
| Pyro-Q | Succinimide |
| Cysteinylation | Amidation |
| Non-classical disulfide linkage | Methionine oxidation |

**Supplementary Table 3.** The information for detection and analysis data of PTMs

| Modification | Control | Low Temp & pH | Combination | Combination /Control (%) |
| --- | --- | --- | --- | --- |
|  | Ratio (%)^f^ | Ratio (%) | Ratio (%) |  |
| Pyro-Q* | 99.4 | 98.8 | 99.0 | 99.6 |
| Succinimide | 7.5 | 7.4 | 5.5 | 73.3 |
| Methionine oxidation | 5.9 | 5.0 | 10.0 | 169.5 |
| Deamidation* | 9.0 | 7.5 | 7.0 | 77.8 |
| Lys-loss+Gly-loss+Amide | 32.3 | 28.0 | 37.2 | 115.2 |
| Lys-loss | 92.4 | 88.0 | 92.2 | 99.8 |
| Sialylation* | / | / | / | / |

**Supplementary Table 4.** Medium Screening to minimize GSH Capping

| Basal Media | Feed Media | Titer  (g/L) | CEX (%) | | | | | | | |
| --- | --- | --- | --- | --- | --- | --- | --- | --- | --- | --- |
|  |  |  | Acidic Peak | | | Main Peak | Basic Peak | | | Acidic Peaks |
|  |  |  | Peak1 | Peak2 | Peak3 | Peak4 | Peak5 | Peak6 | Peak7 |  |
| Actipro | CB7a/7b | 6.53 | 31.7 | 20.3 | 13.2 | 16.9 | 4.6 | 6.0 | 7.4 | 65.2 |
| Vega CHO | AF72V2/CDFS36 | 5.14 | 43.5 | 28.6 | 8.4 | 11.7 | 2.4 | 2.4 | 3.8 | 80.5 |
| AM 08 | AF72V2/CDFS36 | 5.45 | 29.0 | 30.8 | 8.3 | 20.4 | 5.7 | 5.7 | 3.7 | 68.1 |
| Max A | Max FA/FB | 4.32 | 34.9 | 19.7 | 13.2 | 16.3 | 5.8 | 5.8 | 5.8 | 67.8 |
| Max X | Max FX/FB | 5.55 | 58.8 | 17.9 | 8.8 | 6.2 | 2.0 | 2.0 | 4.2 | 85.5 |
